# Do Noncoding and Coding Sites in Angiosperm Chloroplast DNA Have Different Mutation Processes?

**DOI:** 10.3390/genes14010148

**Published:** 2023-01-05

**Authors:** Brian R. Morton

**Affiliations:** Department of Biology, Barnard College, Columbia University, 3009 Broadway, New York, NY 10027, USA; bmorton@barnard.edu

**Keywords:** chloroplast DNA, nucleotide substitution, context, genome evolution

## Abstract

Fourfold degenerate sites within coding regions and intergenic sites have both been used as estimates of neutral evolution. In chloroplast DNA, the pattern of substitution at intergenic sites is strongly dependent on the composition of the surrounding hexanucleotide composed of the three base pairs on each side, which suggests that the mutation process is highly context-dependent in this genome. This study examines the context-dependency of substitutions at fourfold degenerate sites in protein-coding regions and compares the pattern to what has been observed at intergenic sites. Overall, there is strong similarity between the two types of sites, but there are some intriguing differences. One of these is that substitutions of G and C are significantly higher at fourfold degenerate sites across a range of contexts. In fact, A → T and T → A substitutions are the only substitution types that occur at a lower rate at fourfold degenerate sites. The data are not consistent with selective constraints being responsible for the difference in substitution patterns between intergenic and fourfold degenerate sites. Rather, it is suggested that the difference may be a result of different epigenetic modifications that result in slightly different mutation patterns in coding and intergenic DNA.

## 1. Introduction

Since the introduction of the neutral theory [1,2], base composition and substitution rates of noncoding sequences have been used as a proxy for neutral evolution. This provides one mechanism to study the role of selection on putatively neutral substitutions within coding regions, particularly silent substitutions and any associated codon usage bias [1,3,4]. Although there is ample evidence for selection on some noncoding sequences [5,6], the use of intergenic sequence data to estimate neutral evolution assumes that the underlying mutation process is the same across all sites regardless of any functional property of the site, such as coding for an amino acid sequence, which should only affect the subsequent fixation process through selection.

Some recent work has raised questions about this long-held assumption about mutations. Evidence was reported for a decreased mutation rate in exons relative to introns in human DNA [4] and, although this was not supported by an analysis of de novo mutations in germline cells [7], it raises an important question for genome evolution. Although it is not clear what could cause such a difference, it needs to be better established whether or not there is any such relationship between mutation rate and function. Here, I further address this issue using the flowering plant chloroplast genome (cpDNA). Previous studies have shown that substitution dynamics of intergenic, noncoding (NC) DNA in Angiosperm cpDNA are complex. Most notably, they are strongly context-dependent, with evidence for at least three neighboring bases on each side of any site affecting the process, resulting in a wide range of substitution dynamics across sites [8,9]. These complex dynamics have been used as an estimate of neutral evolution to show that the codon usage bias (CUB) of chloroplast genes appears to be largely driven by mutation bias [10].

In the current analysis, substitutions at fourfold degenerate (FFD) sites in protein-coding sequences of Angiosperm cpDNA are analyzed in a context-dependent manner and then compared to what has been observed previously in NC regions. There is no reason to expect a priori that selective constraints across sites are associated with either context or type of mutation (e.g., transition or transversion), so the hypothesis is that variation in context-dependent substitution dynamics is the same across both FFD and NC sites. Any influence of selection is expected to affect only the overall substitution rate, so any other type of variation would be attributable to variation in mutation dynamics. Here, I include repair processes, such as mismatch repair, as part of mutation dynamics, so that mutation includes the phenomena that play out within an individual before the population-level selection process occurs. The data show that FFD and NC sites display different substitution dynamics, while context effects are essentially the same at FFD sites as in NC regions; there are two small but consistent differences between the two types of sites. First, the rate of substitution is greater at FFD sites with the exception of one specific type of substitution: A → T and T → A (W → W) transversions. Second, the predicted equilibrium A+T content is higher at FFD sites in a majority of contexts, and this is matched by observed compositional differences.

Possible neutral explanations for this difference between FFD and NC sites are explored. One is that there is an influence of transcription on mutations, and a second is that there are subtle context effects from a broader neighboring region than the tetranucleotide analyzed in this study, which could be causing the observed differences in substitution as a result of average context differences between FFD and NC sites. Although transcription appears to have some effect on substitution rate, the data do not support either of these two mechanisms explaining the difference between FFD and NC sites. The third possibility is that the mutation process is not quite equivalent at the two site types. This does not require a difference in mutation dynamics resulting directly from function. Rather, it could be a consequence of some other difference that is related to functionality and/or expression. The evidence shows that there is a potential CG effect—an increased rate of C → T substitutions upstream of a G that could arise from a high rate of deamination of methylated Cs—and although it is shown that any CG effect cannot account for the difference between FFD and NC sites, it is proposed that the difference between FFD and NC sites might be due to differences in epigenetic modifications that give rise to variation in mutation dynamics across selectively neutral sites. Regardless of the specific reason, though, the results indicate that there are complications to the application of NC data in analyses of selection on coding sequences.

## 2. Materials and Methods

The analysis of substitutions followed the methodology described previously for noncoding DNA regions [9]. RefSeq complete Angiosperm chloroplast genome sequences were downloaded from NCBI www.ncbi.nlm.nih.gov/genome/browse#!/eukaryotes/ (accessed on 14 March 2019) and then parsed with the Biopython 1.76 [11]. Genomes were grouped into 280 closely related triplets from 40 different families, which were chosen to ensure non-overlapping lineages, and the coding strand of each CDS was then aligned using the *pairwise* function from Biopython with the parameters set as *match* = 2, *mismatch* = −1, *gap open* = −2, and *gap extend* = −0.5. Any CDS alignment with more than 30 total gaps introduced was excluded from further analysis.

The alignments were used to generate 128 tetranucleotide context-dependent substitution matrices, one matrix for each of the tetranucleotide contexts within which a FFD site can occur (e.g., no FFD site can have a 5′ A due to the genetic code.) Any substitution site N_0_ can be considered as the central site of the pentanucleotide {N_2_ N_1_ [N_0_] N_1_ N_2_} where N_1_, and N_2_ are the neighboring base pairs that compose the tetranucleotide context flanking N_0_. To determine the codon position of any N_0_ site, it was assumed that any gaps introduced during alignment did not change the reading frame, so positions that were a multiple of three were assumed to be potential FFD sites (which is determined by the content of the 5′ N_2_ and N_1_ nucleotides). Count Matrices were generated and then the complementary matrices combined, following which Rate Matrices (also Markov Transition matrices) and the stationary vector for each matrix were all performed as described previously [9]. All comparisons were between the combined strand matrices, or the matrices generated by combining complementary pairs. To estimate the sampling error for the A+T content of the stationary vector, a bootstrap method was employed. Each resample generated a matrix with the same number of off-diagonals, or substitutions, in each row with the substitutions drawn from the matrix row with the replacement. The A+T content of the stationary vector from this resampled matrix was calculated, and the average and standard deviation determined for 1000 resamples. Substitutions within tetranucleotide contexts from noncoding regions were taken from the previous analysis [9]. All calculations were done using Python script written by the author.

## 3. Results

### 3.1. Comparison of Substitution Rates at FFD and NC Sites

Substitutions at FFD sites in Angiosperm coding regions were scored to generate 128 tetranucleotide context-dependent matrices, one for each context that can occur for FFD coding strand sites given the genetic code. The analysis used the same set of 280 taxa triplets that were used in the intergenic (NC) DNA study [9], so the total evolutionary time is identical for the two. Contexts were restricted to the surrounding tetranucleotide, instead of the full hexanucleotide contexts that were analyzed for the NC data, since many hexanucleotide contexts had fewer than 10 total substitutions in the FFD data. There were 357,783 substitutions observed across 4,480,496 FFD sites, which averages to 2795.2 substitutions/matrix and 0.080 substitutions/site. As in the NC analysis, multiple hits were ignored given the low substitutions per site, and so the substitutions/site will be referred to as the rate of substitution. Once complementary matrices were combined, as described in the Materials and Methods Section, there were 689,424 substitutions at 8,706,350 FFD sites. In these combined matrices, there are 192 contexts for which there can be substitution data matrices given the genetic code, yielding an average of 3590.8 substitutions/matrix. Since complementary matrices are redundant, 100 of these 192 matrices are unique and can be used in an analysis.

**Figure 1:**
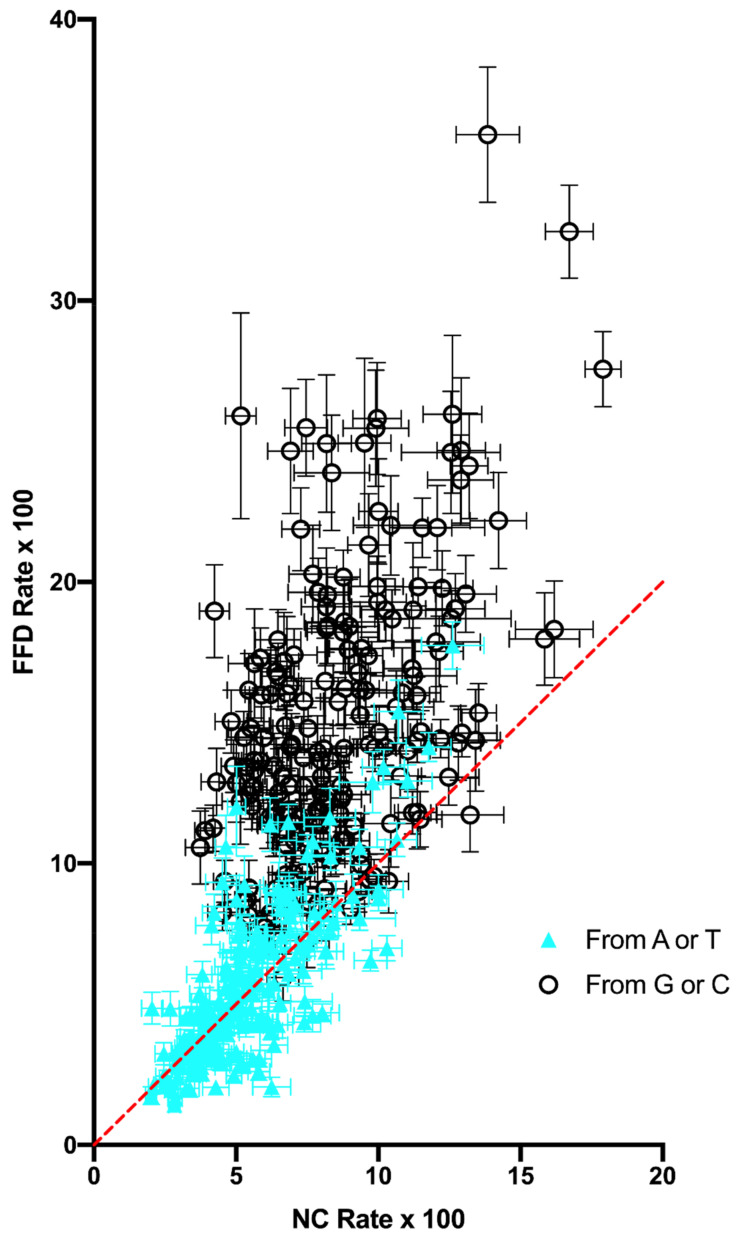
Rate of substitution of FFD and NC sites of each nucleotide within each tetranucleotide context. Rate for each matrix row is calculated as the number of off-diagonals in that row divided by the row total. Substitution rates from A or T (closed blue triangles) are higher in FFD in 96 of 192 contexts, while substitution rates from G or C (black circles) are higher in FFD in 184 of 192 contexts. The equality line is drawn in red.

A comparison between FFD and NC sites of the rate of substitution of each individual base within each specific tetranucleotide context (i.e., the individual rows within each matrix) is shown in Figure 1. A comparison of the total rate, as opposed to individual nucleotides, across each context shows the same general trend (Supplemental Figure S1: Substitution Rate Comparison). Variation is highly correlated across contexts (r^2^ = 0.521), indicating that the effect of context is similar in the two types of putatively neutral sites. Despite the similar trend, though, it is notable that FFD sites have a higher substitution rate in 72.9% of the cases (substitution of a specific base within a specific context). In those cases where FFD sites have a higher substitution rate, the rate is 69.3% greater on average than the NC rate. In the remaining cases (27.1%), where the NC sites have a higher rate, they are only 21.7% greater, on average, than the FFD rate. As is apparent in Figure 1, the higher average rate at FFD sites is largely the result of a higher general rate of substitution of G and C. When the substituted base is A or T, FFD sites have a higher rate than NC sites in only 50% of the cases, with a Bayes Factor = 0.18 [12], assuming an equal probability of FFD and NC having a higher rate, while when a G or C is being substituted, FFD sites are greater in 95.8% of the cases (Bayes Factor >> 10^6^). There is also a stronger correlation between NC and FFD contexts for substitutions from A or T (r^2^ = 0.592) than from G or C (r^2^ = 0.242).

**Figure 2:**
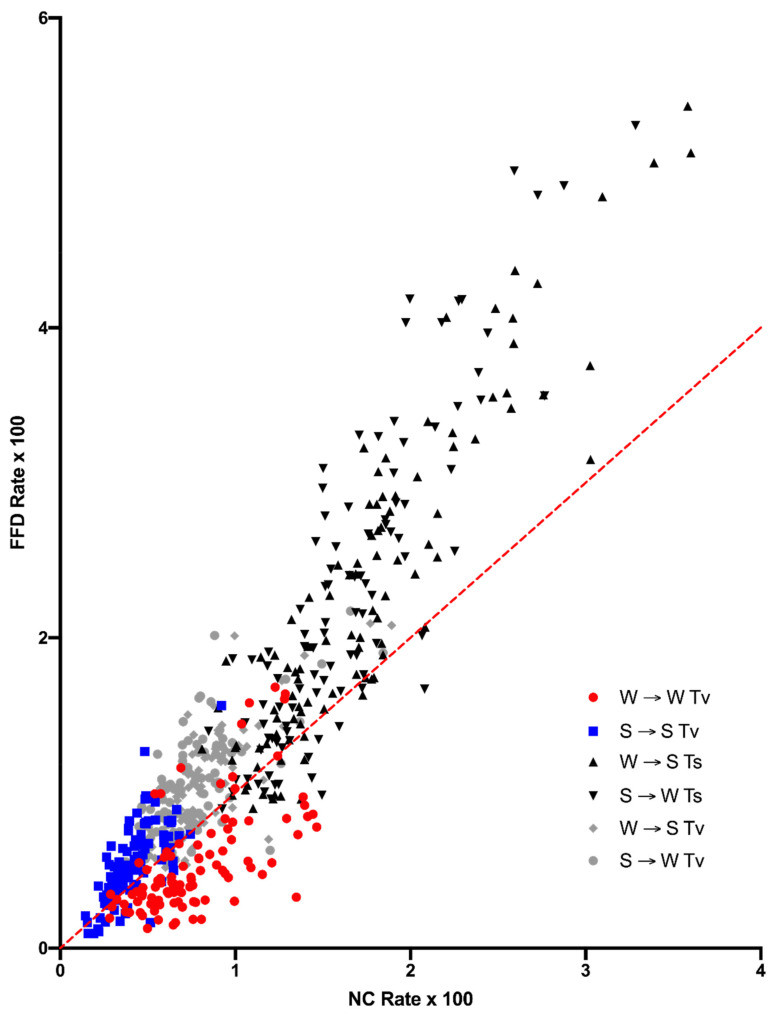
Rates of specific mutation types compared between NC and FFD sites, with the rate calculated as in Figure 1 and the equality line in red. Confidence intervals are omitted for clarity. The W-W substitutions (red circles), which are the A → T and T → A transversions, are below the equality line in 85% of the cases, while all others are above the equality line in about 87% of the cases.

The difference in substitution rate between FFD and NC sites in Figure 1 is quite different for the various types of substitutions. Specifically, one type of substitution displays a pattern that is opposite of what is observed for other substitutions. A/T(W) → G/C(S) and S → W transitions are higher at FFD sites in 85% and 84% of the contexts, respectively, and W → S, S → W, and S → S transversions are higher in 87%, 91%, and 78% of contexts, respectively. The exception is the case of the W → W (A → T and T → A) transversions, which are higher at FFD sites in only 15% of the contexts (Figure 2). A comparison of the proportion to a 50% chance that FFD > NC in that context yields a Bayes Factor greater than 10^6^ in each case. Thus, any explanation of the difference in substitutions between FFD and NC sites needs to account for the fact that W → W transversions show a higher rate at NC sites, while all other substitution types are higher at FFD sites.

### 3.2. Comparison of Equilibrium Compositions of FFD and NC Sites

**Figure 3:**
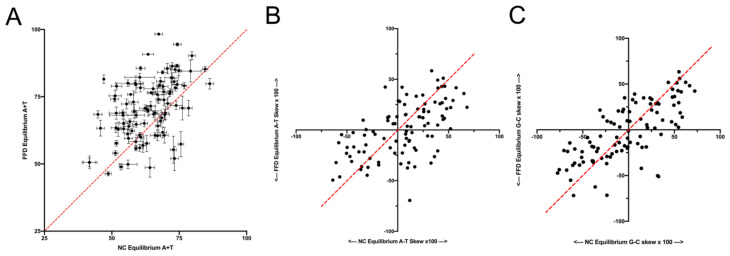
Comparison of FFD and NC sites in the equilibrium composition predicted by the substitution dynamics in each context. (**A**) Predicted A+T content, (**B**) predicted A-T skew ((A−T)/(A+T)) at equilibrium and (**C**) predicted G-C skew ((G−C)/(G+C)). For the predicted A+T content, error bars were determined using the resampling methodology described in the Materials and Methods Section.

The full substitution rate matrix for each context can be used to calculate a stationary vector, which represents the base composition that would exist at those sites at evolutionary equilibrium. Although the context of a site will change over time, differences in the equilibrium vector represent differences in the evolutionary trajectories across contexts, and this provides another way to study variation in substitution dynamics. A comparison of the equilibrium base compositions that are predicted to evolve across contexts finds that there is a difference between FFD and NC sites. In 73% of the tetranucleotide contexts, the substitution dynamics at FFD sites would lead to a higher equilibrium A+T content than the substitution dynamics of NC sites in the same context (Figure 3A), with an average absolute difference of 10.1% A+T. This proportion is compared to a 50% chance that FFD > NC in any given context yields a Bayes Factor of 5 × 10^3^ [12]. This higher average A+T content appears to be consistent with the higher rate of substitution of G and C nucleotides at FFD sites that was described above. Despite the difference in A+T content, the predicted skews at equilibrium do not appear to differ between FFD and NC sites, with both G-C and A-T skews being distributed around the equality line (Figure 3B,C).

**Figure 4:**
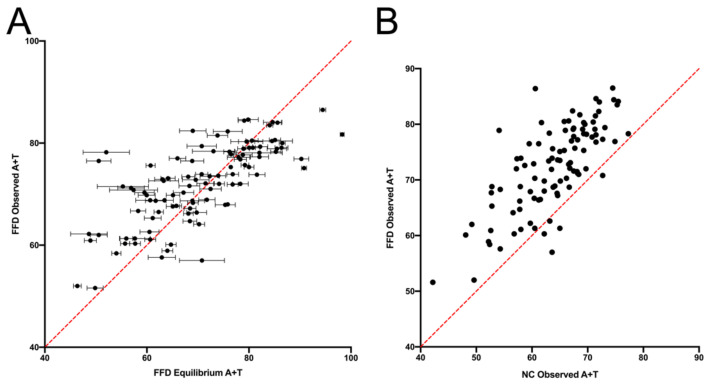
A comparison of (**A**) the predicted and observed A+T content at FFD sites and (**B**) the observed A+T content at NC and FFD sites. For the predicted A+T content of FFD sites, error bars were determined using the resampling methodology described in the Materials and Methods Section.

The prediction that FFD sites will evolve to a slightly higher A+T content than NC regions is consistent with the observed base composition. The context-dependent A+T content is correlated with the equilibrium predictions for FFD sites (r^2^ = 0.538, Figure 4A), as was the case for NC sites [9], and the observed context-dependent A+T contents of FFD and NC sites differ in the same way that the predicted compositions do (Figure 4B). These data, along with the difference in rates observed in Figure 1, strongly suggest that there is some underlying difference in substitution dynamics at FFD and NC sites that generates different average evolutionary trajectories.

### 3.3. What Accounts for the Difference between FFD and NC Sites?

One possible explanation for the difference is that selective pressures or constraints differ between FFD and NC sites. This will be examined below in the Discussion Section. Here, we examine three potential neutral explanations for why NC and FFD sites might show different substitution dynamics.

#### 3.3.1. Transcription

**Figure 5:**
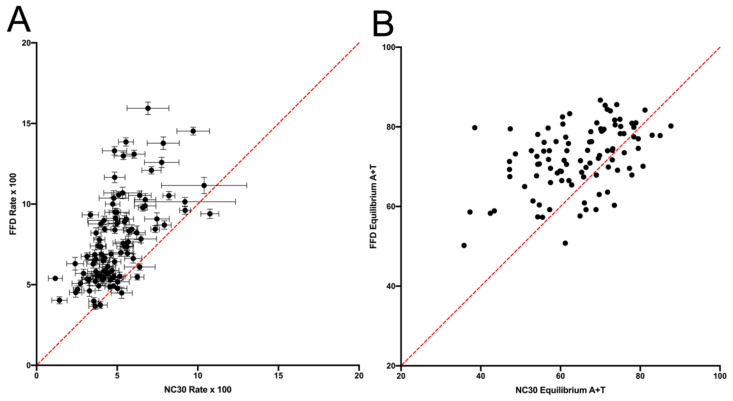
Comparison of (**A**) substitution rate and (**B**) predicted equilibrium A+T content for FFD sites and NC sites within 30 nucleotides of a start or stop codon (NC30 sites).

An association between transcription and repair has long been known [13] and can affect genome composition patterns [14]. To test the influence of transcription in this analysis, the NC data were reanalyzed using only those sites within 30 nucleotides of a start or stop codon, called here the NC30 dataset. Although we lack accurate transcript maps for all chloroplast genomes, the assumption is that the NC30 dataset is made up predominantly of noncoding sites that are transcribed. Although the equilibrium A+T values are consistent for NC30 and NC sites (Supplemental Figure S2: Equilibrium A+T of NC30 and NC Sites), there are rate differences between the NC30 and NC data, indicating that transcription might influence substitution rates to some degree (Supplemental Figure S3: Rate Comparison Between NC and NC30 Sites). However, the differences that are observed between FFD and NC sites are also observed when we compare the FFD and NC30 (Figure 5). FFD sites have a higher rate than NC30 sites in 94% of the contexts and 78% of the contexts have a higher predicted equilibrium A+T content at FFD than at NC30 sites, with an average difference of 10.3%. These results do not support the hypothesis that transcription effects could explain the difference in substitutions between FFD and NC sites, although accurate mapping of transcripts in the future would allow us to test this hypothesis more precisely.

#### 3.3.2. Inferred Hexanucleotide Context Effects

Another possible neutral explanation for the difference in substitution dynamics between FFD and NC sites is an influence on mutations of nucleotides beyond the tetranucleotide context. The analysis of NC regions indicates that the sites that are three nucleotides removed from the substitution, which in combination with the tetranucleotide context would form the hexanucleotide context, do have an effect on substitutions [9]. Although the nucleotides that are three sites removed from an FFD site are both third codon position nucleotides, which are A+T-rich in cpDNA, such as NC regions [3], FFD, and NC sites that are in the same tetranucleotide context, might occur in a different distribution of hexanucleotide contexts. This could in turn result in different substitution dynamics across tetranucleotide contexts.

Since the FFD dataset was limited to tetranucleotide contexts due to sample size, the hypothesis was tested by calculating a weighted predicted A+T (AT_w_) content for each FFD tetranucleotide context and a weighted rate (R_w_) for each nucleotide within each context. For each tetranucleotide context, AT_w_ was determined using a weighted average of the equilibrium A+T values from the NC data [9] as follows. For any tetranucleotide context, there are 16 hexanucleotide contexts that contain that internal tetranucleotide (i.e., the 16 nucleotide pairs of the two bases that are three sites removed from the substitution). AT_w_ was calculated as the average of the NC equilibrium A+T content of these 16 hexanucleotide contexts, weighted by the observed frequency of that hexanucleotide among all FFD sites in the same tetranucleotide context. The same method was applied to calculate R_w_ values. These yield values are expected at sites that are following NC substitution dynamics but distributed across hexanucleotide contexts in the same way that the FFD sites are. Thus, if the difference between FFD and NC sites is due to a different distribution within hexanucleotide contexts then the FFD sites should match the weighted values.

**Figure 6:**
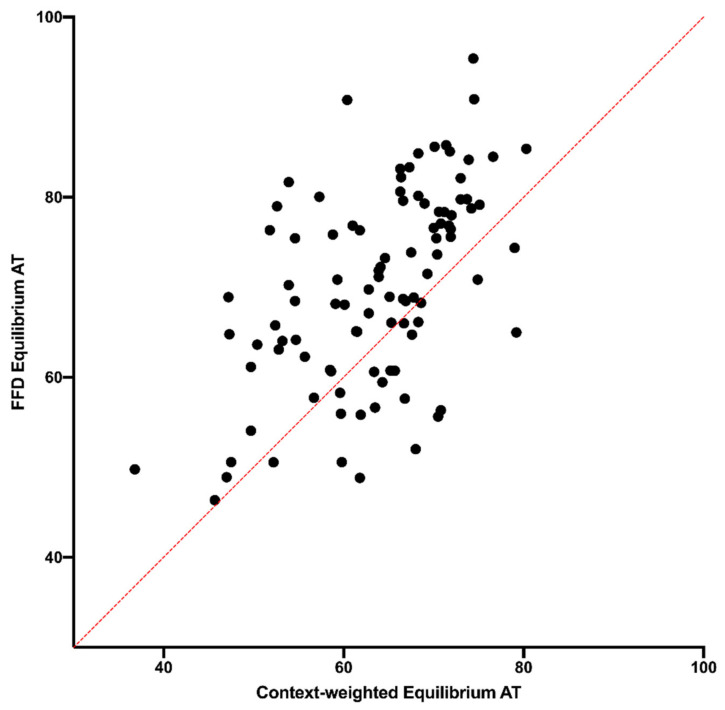
A comparison of predicted equilibrium A+T content at FFD sites and the AT_w_ values, which are weighted by context distribution as described in the text.

**Figure 7:**
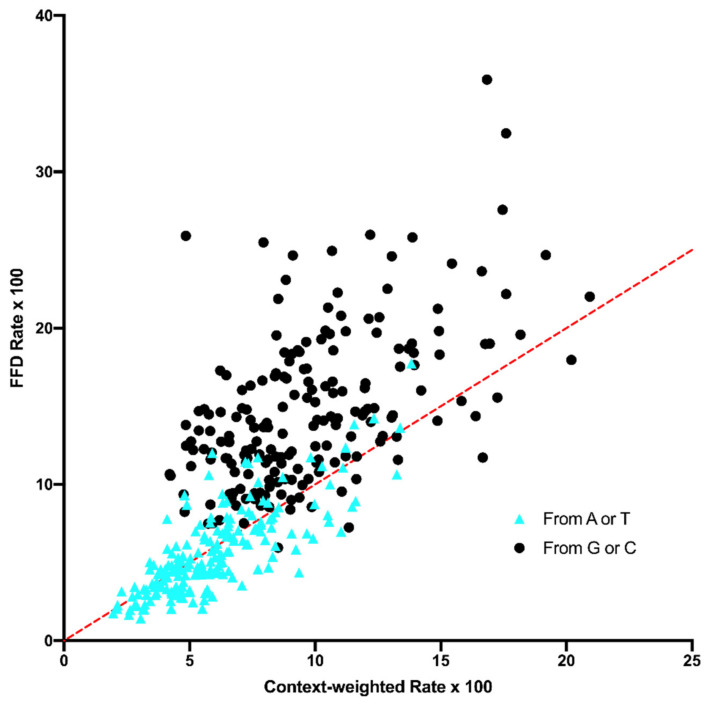
A comparison of context-dependent substitution rates at FFD sites to the R_w_ values, which are weighted by context distribution as described in the text. Substitutions of A or T are shown as a blue triangle.

Using the observed hexanucleotide frequencies from the sequences of all cpDNA genes in the taxa—*Zea mays* and *Zingiber officinale*, *Lillium lancifolium*, *Prunus salicina*, and *Brassica napus,* which represent a range of monocots and dicots—AT_w_ was calculated and compared to the equilibrium A+T of FFD sites (Figure 6), and R_w_ was calculated and compared to FFD rates (Figure 7). Sampling error bars are excluded to maintain simplicity. The data do not support the hypothesis that a different distribution across hexanucleotide contexts is responsible the difference between NC and FFD sites in either substitution rate or equilibrium A+T content across tetranucleotide contexts. In the comparison of A+T content, 77.8% of contexts have a greater equilibrium A+T at FFD than expected (Bayes Factor = 9.4 × 10^5^), and in the case of rates, 252 of 384 (65.2%) contexts have a greater than expected rate with a Bayes Factor of 17.4 × 10^6^. These data indicate that there are real differences in substitution dynamics between NC and FFD sites that yield different rates and equilibrium A+T contents across contexts. 

#### 3.3.3. Epigenetic Effects such as CG Deamination

A third possible neutral explanation is that there are differences between FFD and NC sites in the types and/or levels of epigenetic modification. Epigenetic modification can lead to changes in the rate of specific mutations, with the most commonly studied example being an increased rate of mutation at methylated CG sites as a result of deamination, sometimes called the CG effect [15,16,17,18]. There is evidence for CpG methylation in cpDNA [19], and the data from the NC regions were consistent with a CG effect [9], which raises the possibility that differences in methylation levels, or some other epigenetic modification, could explain the differences between substitutions at FFD and NC sites.

**Figure 8:**
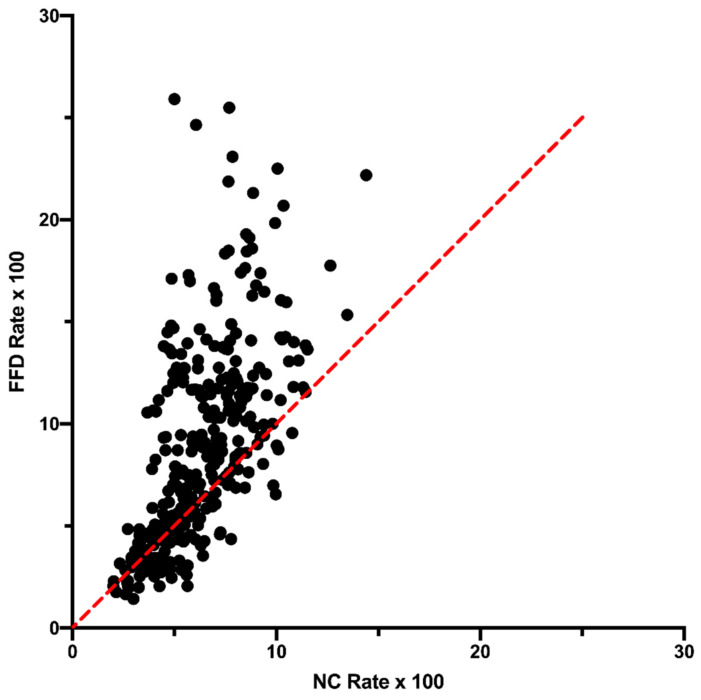
Comparison of context-dependent substitution rate excluding all sites with a potential mutation of a CG dinucleotide. Error bars are excluded for clarity.

The data here are consistent with a CG effect in FFD sites (Table 1), and the roughly 42% increase of transitions in CG contexts is very similar to the 37% increase observed in noncoding DNA [9]. When we exclude those cases in which there is a potential CG substitution (i.e., {N_2_ N_1_ C G N_2_} and {N_2_ C G N_1_ N_2_} pentanucleotides using the notation in the Materials and Methods Section with the substitution site underlined), the differences between FFD and NC sites that are seen in Figure 1 are still present (Figure 8). Thus, the data indicate that epigenetic modifications can influence the underlying mutation dynamics at a site. While it does not appear that a CG effect is responsible for the differences between FFD and NC sites, the data do not exclude the possibility that there are other types of base modifications in cpDNA and that these could explain the differences between FFD and NC substitutions if they occur at different frequencies in coding and noncoding DNA.

## 4. Discussion

This study compared substitutions at fourfold degenerate (FFD) coding sites and noncoding (NC) intergenic sites, controlling for the composition of the tetranucleotide surrounding each substitution, or context-dependent effects [9]. The expectation is that, if mutation dynamics are the same at FFD and NC sites, then they should display the same context-dependent substitution dynamics in the absence of selection. It is shown that, although variation across sites is strongly correlated between the FFD and NC data, the results strongly suggest that there is some difference in substitution dynamics between them. The data show both that FFD sites have a higher rate of substitution, with the exception of W → W transversions, which have a higher rate at NC sites, and that the two types of sites differ in their substitution dynamics in some manner such that FFD sites are expected to evolve to a higher equilibrium A+T content, a prediction that is supported by compositional data. These two observations, which come from different ways of comparing the substitution data, are likely to be related in some way.

The most obvious possible explanation for the observed differences in substitution dynamics is that there are selective differences between NC and FFD. However, it is difficult to develop a selection model that could explain the data. There could be selective constraints on FFD sites, such as codon adaptation [3,20], effects of codon usage on protein folding [21], and/or mRNA secondary structure [22], but these would be expected to decrease the substitution rate, not increase it, relative to NC sites. The higher general rate of G and C substitutions at FFD sites could indicate positive selection for A and T or, conversely, selection against these bases at NC sites. However, the fact that NC sites have a lower rate of T → A and A → T transversions (Figure 2) is not consistent with selection is simply for A+T content, nor is the difference in W → S and S → W transversions between the two site types. There is no obvious selection model to account for the increased rate of substitution at FFD sites with the sole exception of W → W transversions, which are actually decreased relative to NC sites.

A second difficulty with a selective explanation is that codon adaptation in flowering plant chloroplast genes is apparent in the atypical codon usage of the highly expressed *psbA* gene, but there is no evidence for widespread adaptation across genes, which shows a general bias towards A and T at the synonymous positions [3,23]. Thus, selection for higher rates of G and C substitution would need to be the result of something other than selection for translation efficiency, either another form of selection on codon usage or on some factor other than codon usage, such as mRNA stability and influences on protein folding. It has long been established that Angiosperm chloroplasts have a low effective population size [24] such that cpDNA is influenced by drift to a much higher degree than the nucleus [25]. So, the substitution data would require either strong positive selection on mutations, with the exception of W → W transversions in coding regions, or selection against everything except these transversions in NC regions. Such strong selection would run counter to the observations about codon usage in cpDNA [23].

While a selective explanation cannot be excluded, there is no adequate model to explain the substitution data here. In the absence of a good selective explanation, we considered possible neutral explanations that would be based on some difference in mutation dynamics between FFD and NC sites. This would not need to be a direct difference that results from functionality of a site, and there is no model for how such a difference could exist, but instead could be an indirect result of some difference that arises because of the functional differences.

One neutral explanation could be that there is a difference in transcription level between the site types and this affects some aspect of mutation/repair [13,14]. This would be similar to the possibility that a difference in mutation and repair dynamics between leading and lagging strand contributes to strand asymmetry in some bacterial genomes [26]. The data from the NC30 analysis here show that, although that transcription might influence substitution dynamics to some degree, any effect cannot account for the difference between FFD and NC sites. However, a fuller examination of this possibility should be performed based on transcript mapping and levels. A second neutral explanation could be that the influence of context on mutation is more complex than what was controlled for. Even though the data indicate that the difference between FFD and NC sites exists when controlling for the composition of the surrounding hexanucleotide, there could be subtle effects of an even broader region, which, on average, differ in composition between coding and noncoding sites. The data in Figure 6 and Figure 7 suggest that wider context effects do not account for the difference between FFD and NC sites. This is perhaps not surprising since, as described in the Materials and Methods Section, the pentanucleotide {N_2_ N_1_ [N_0_] N_1_ N_2_} is composed of the N_1_ and N_2_ neighboring base pairs that compose the tetranucleotide context flanking the site of substitution N_0_. If we expand this to the heptanucleotide {N_3_ N_2_ N_1_ [N_0_] N_1_ N_2_ N_3_}, where the N_0_ substitution site is FFD, then the two bases that make up the N_3_ neighboring pair would both be third codon position sites, which are generally A+T-rich like intergenic regions. Thus, the distribution of tetranucleotide contexts across hexanucleotide contexts are probably fairly similar for FFD and NC sites.

A third potential neutral explanation is that there are differences in epigenetic modification that give rise to differences in rates of specific types of mutation across sites, such as what we observe in the deamination of methylated CG sites [15,16,17,18]. The similarity in the increased rate of CT transitions in the CG context here (Table 1) does not support any effect of CG methylation on the difference between FFD and NC sites. However, it is possible that there are other types of modification, such as CNG methylation, in cpDNA that differ between coding and noncoding DNA and which could give rise to a difference in underlying mutation bias. Particularly due to the difference between W → W and other types of substitution, this appears to be the most promising hypothesis to explain the substitution data in this study and should be explored in future analyses. It could also explain the rate differences between NC30 and NC sites (Supplemental Figure S3) if these sites tend to differ in epigenetic modifications due to proximity to coding DNA.

Regardless of the underlying reason for the differences in substitution dynamics between FFD and NC sites, the observations here raise some caution for the use of either type of site as a proxy for neutral evolution. A comparison of exon and intron SNPs from the human genome suggested a similar phenomenon [4], and although the results were not replicated using germline mutation data [7], some profit should come from more thoroughly exploring the factors that influence mutation dynamics in various genomes. Of particular interest here, substitution data from NC sites have been used to explain codon usage bias in flowering plant genes, with the results indicating that selection is not necessary to explain codon usage except in the case of the *psbA* gene [10]. The basis of this was basically the fact that the composition of synonymous sites in coding genes was consistent with expected compositions generated using the context-dependent substitution patterns in intergenic regions. This is intriguing given the main findings here. Since the difference between FFD and NC sites is predominant in substitutions of G and C, and silent sites in chloroplast genes are strongly A+T-rich, it is quite possible that any difference between FFD and NC sites is too small to affect the use of NC substitutions to predict codon bias. However, further analysis is necessary to assess the use of intergenic regions to predict neutral composition patterns within coding sequences.

## Figures and Tables

**Table 1:** A comparison of the rate of C → T substitution within CG dinucleotides ^1^ to the C → T rate in other contexts.

	Substitution ^2^			Substitution	
	G → A	G → B		C → T	C → V
CG:	34 376	304 753	CG:	35 584	320 038
DG:	55 100	699 456	CH:	54 738	695 595
	OR = 1.43			OR = 1.41	

^1^. Substitutions are scored from C, either a transition or a transversion, on either strand of DNA. The substituted base within the CG nucleotide is underlined in each case. ^2^. D = A, G, or T (i.e., Not C); B = Not A; V = Not T; H = Not G.

## Data Availability

The FFD matrices are available at Dryad (https://doi.org/10.5061/dryad.f7m0cfz1b, accessed on 2 January 2023).

## Supplementary Materials

The following supporting information can be downloaded at: https://www.mdpi.com/article/10.3390/genes14010148/s1, Figure S1: Substitution Rate Comparison, Figure S2: Equilibrium A+T of NC30 and NC Sites, Figure S3: Rate Comparison Between NC and NC30 Sites.
