# Supplementary material for: Do Noncoding and Coding Sites in Angiosperm Chloroplast DNA Have Different Mutation Processes?
Source: Genes (Basel). 2023 Jan 5;14(1):148. doi: 10.3390/genes14010148 (PMC9858945; doi:10.3390/genes14010148)
Supplement: Supplementary file 1 [file genes-14-00148-s001.zip › genes-2101390-supplementary.pdf]

Supplemental Figure S1: Substitution Rate Comparison

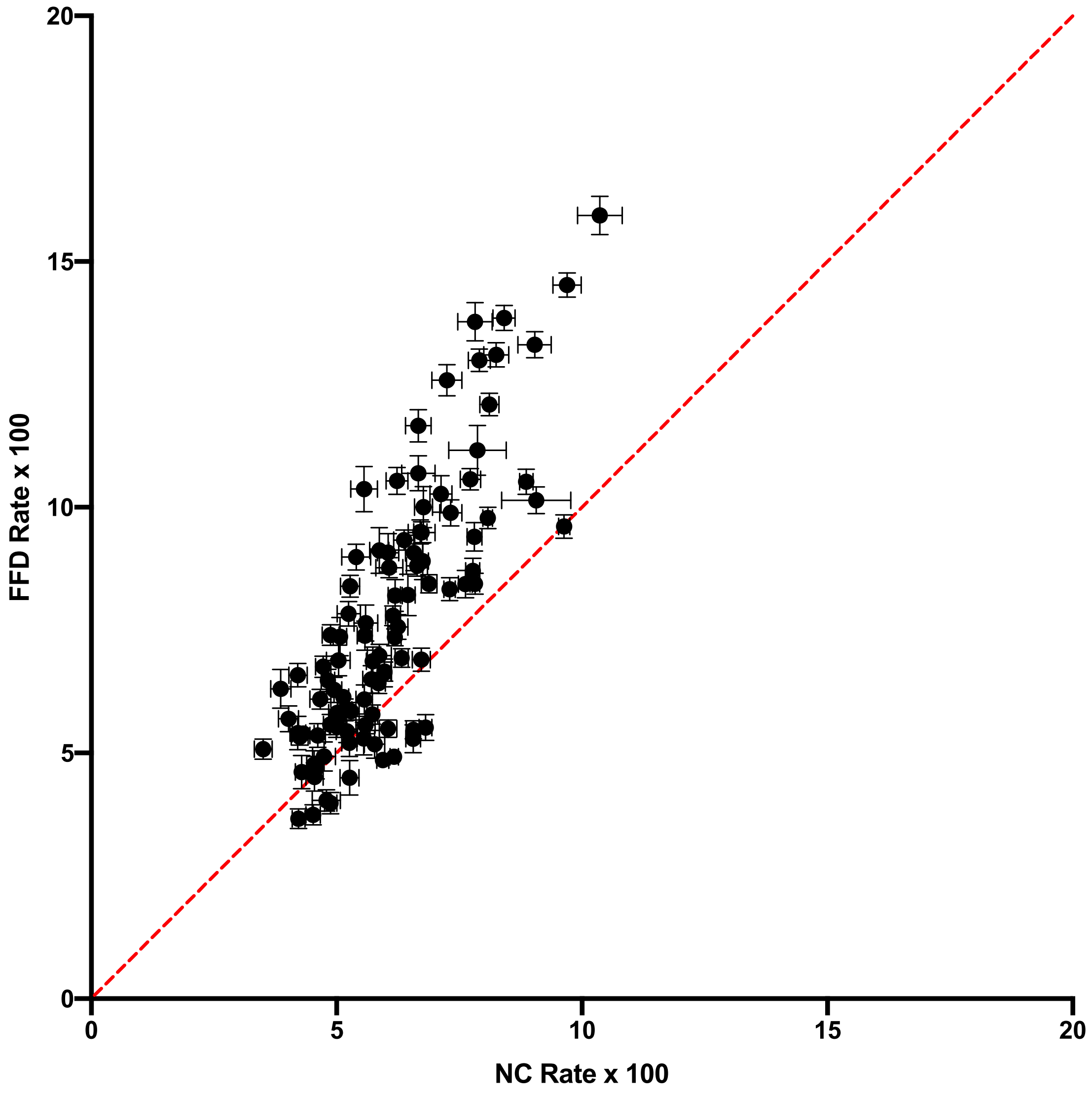

A comparison of context-dependent substitution rate at FFD and NC sites. Each point represents the full rate in one tetranucleotide context.

Supplemental Figure S2: Equilibrium A+T of NC30 and NC Sites

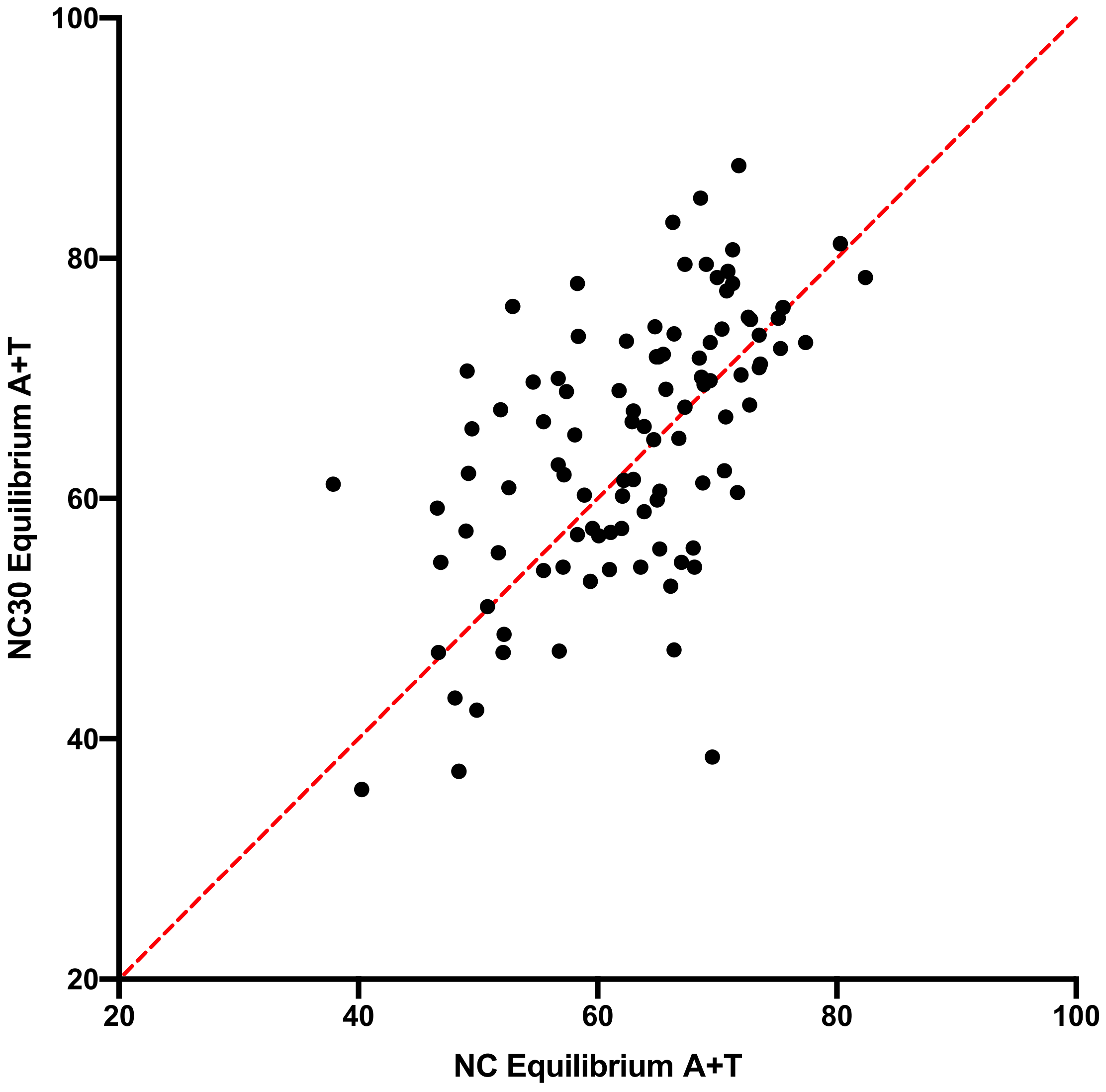

A comparison of predicted equilibrium A+T for NC30 sites and all other NC sites.

Supplemental Figure S3: Rate Comparison Between NC and NC30 Sites

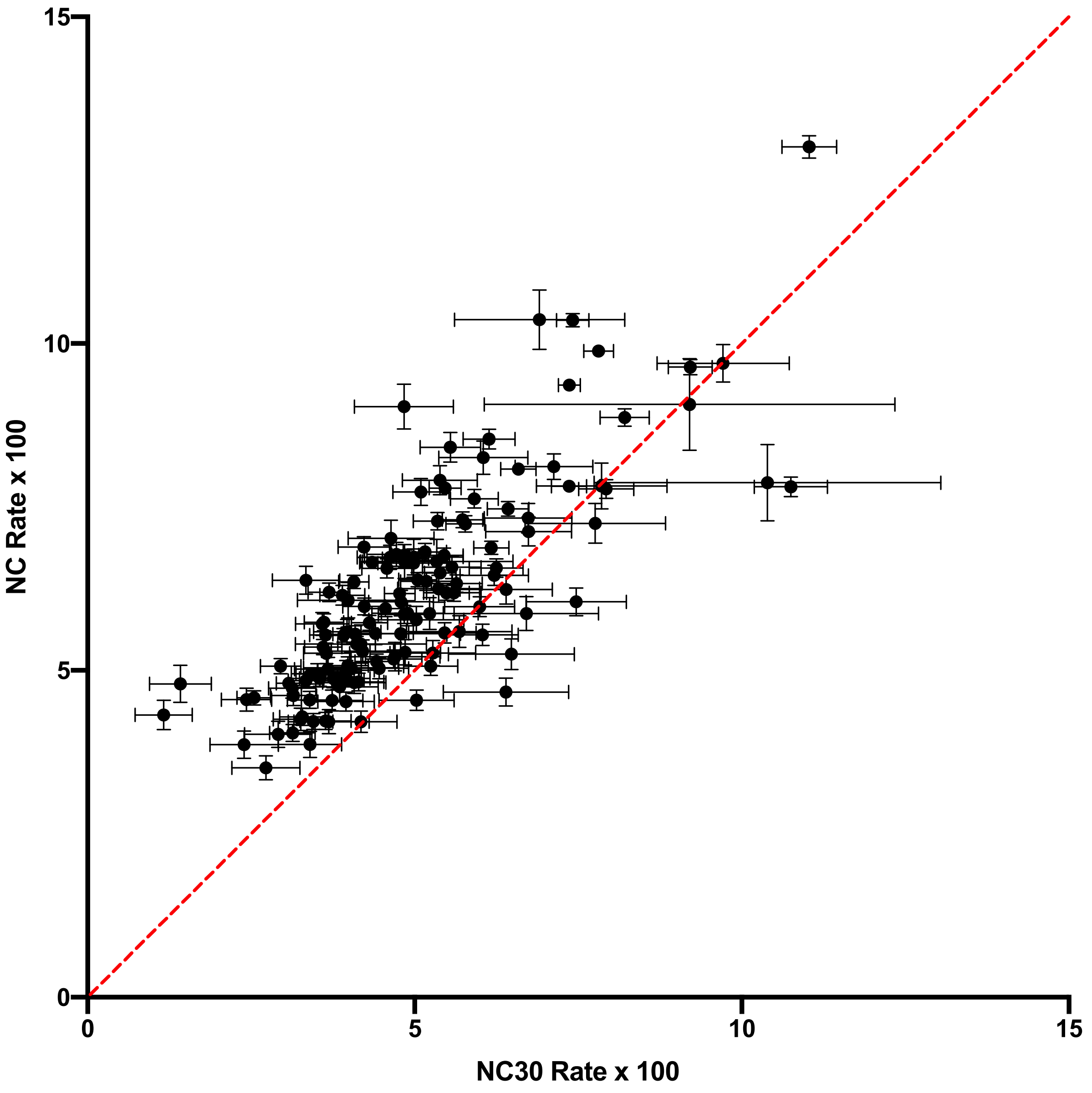

A comparison of substitution rate across contexts for NC30 sites and all other NC sites.
